# Associations between grip strength and glycemic control in type 2 diabetes mellitus: an analysis of data from the 2014-2019 Korea National Health and Nutrition Examination Survey

**DOI:** 10.4178/epih.e2021080

**Published:** 2021-10-08

**Authors:** Harim Choe, Hoyong Sung, Geon Hui Kim, On Lee, Hyo Youl Moon, Yeon Soo Kim

**Affiliations:** 1 Department of Physical Education, College of Education, Seoul National University, Seoul, Korea; 2 Korea Institute of Sport Science, Seoul, Korea; 3 Institute of Sports Science, Seoul National University, Seoul, Korea

**Keywords:** Hand strength, Muscle strength, Glycated hemoglobin A

## Abstract

**OBJECTIVES:**

Glycemic control is essential for preventing severe complications in patients with diabetes mellitus. This study investigated the association between grip strength and glycemic control in Korean adults with type 2 diabetes mellitus.

**METHODS:**

From the Korea National Health and Nutrition Examination Survey, 2,498 participants aged over 19 years that patients with diabetes mellitus who did not have a history of cardiovascular disease or cancer were selected for analysis. Grip strength was assessed using a handheld dynamometer and was represented as age-specific and sex-specific tertiles. Multivariable logistic regression was performed to calculate the odds ratio (OR) and 95% confidence interval (CI) of glycemic control according to the grip strength tertiles.

**RESULTS:**

A significantly lower probability (OR, 0.67; 95% CI, 0.47 to 0.97) for glycemic control was found in the lowest tertile of grip strength compared to the highest tertile. Furthermore, a subgroup analysis by sex only found significant associations between grip strength and glycemic control in males.

**CONCLUSIONS:**

Lower grip strength was associated with poor glycemic control in patients with diabetes mellitus, especially in males. However, further studies are needed to confirm the causal relationship between grip strength and glycemic control.

## INTRODUCTION

Type 2 diabetes mellitus (T2DM) is a chronic condition characterized by sustained high blood glucose levels, and its complications include cardiovascular disease (CVD), kidney failure, retinopathy, and peripheral vascular disease [1]. The International Diabetes Federation projected that the prevalence of T2DM will be 51% higher in 2045 than in 2019 [1]. T2DM is also a major public health problem in Korea, as its prevalence has increased every year from 2005 to 2015 [2]. In addition, T2DM was the ninth highest cause of global death in 2019, and it was the sixth leading cause of death in Korea [3,4].

Hemoglobin A1c (HbA1c) is used to confirm whether blood glucose has been regularly controlled in patients with diabetes [5,6]. T2DM patients with higher HbA1c levels are at an elevated risk of developing CVD and mortality compared to those with lower HbA1c levels [7,8]. Therefore, patients with diabetes are recommended to carefully control their blood glucose levels to prevent complications [9]. However, although 65.0% of Korean adults with diabetes were aware that they had been diagnosed with diabetes, only 28.3% of patients with diabetes were able to achieve glycemic control [10].

The skeletal muscles are the primary storage site of plasma glucose, which is converted to glycogen, and are the most prominent protein reservoir [11]. In this regard, insulin metabolizes both plasma glucose and protein in skeletal muscle [11,12]. Moreover, muscle strength—a characteristic of skeletal muscles—is an essential component of physical fitness [13] that can only be improved by long-term exercise [14]. Unfortunately, muscle strength decreases with age [15]. As demonstrated in previous studies, muscle strength is inversely associated with the risk of mortality [16], CVD [17], and cancer [18]. Furthermore, patients with diabetes who had lower muscle strength were found to be at a significantly higher risk of mortality and CVD incidence than those with higher muscle strength [19]. Low muscle strength was also associated with deleterious indices, presenting a higher risk of atherosclerosis and diabetic peripheral neuropathy, in patients with diabetes [20,21].

To date, few studies have investigated the association between grip strength and glycemic control in patients with T2DM. Moreover, this relationship has not been examined in Korean adults. Therefore, the present study aimed to determine the association between grip strength and glycemic control in patients with diabetes mellitus.

## MATERIALS AND METHODS

### Study participants

**Figure 1.**
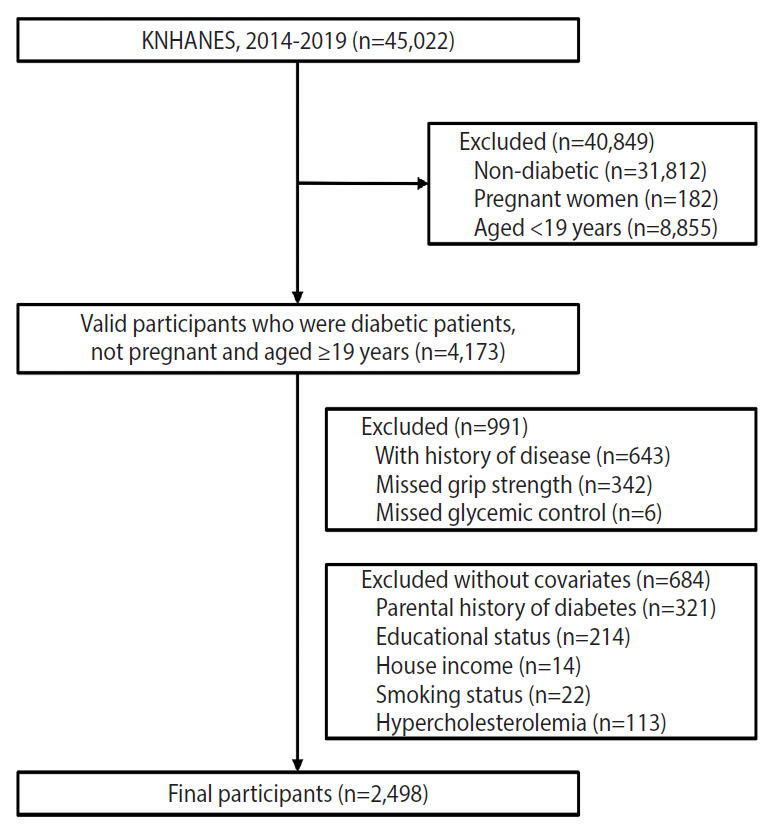
Flow diagram of participants excluded. KNHANES, Korea National Health and Nutrition Examination Survey.

This study analyzed data from the Korea National Health and Nutrition Examination Survey (KNHANES) 2014–2019. The KNHANES is conducted annually by the Korea Centers for Disease Control and Prevention (now known as the Korea Disease Control and Prevention Agency) to collect information about the health behavior, prevalence of chronic diseases, and nutritional status of the Korean population [22]. Specifically, from 2014 to 2019, the KNHANES surveyed a total of 45,022 participants. Individuals with T2DM aged 19 years or older who were not pregnant female were included in the current study (n=4,173). Moreover, participants were excluded if they reported a history of CVD or cancer (n=643) or did not undergo HbA1c or grip strength measurements (n=348). Furthermore, those who had missing data for the covariates (n=684) were excluded. Finally, 2,498 participants were selected for this study (Figure 1).

### Grip strength

Grip strength was assessed with participants in a standing position, without bending the elbow or wrist, and the handle was adjusted to 90° with the second finger joint. Three measurements were made for both hands using a digital handheld dynamometer (T.K.K.5401; Takei Scientific Instruments Co, Ltd, Tokyo, Japan), with 1-minute intervals between trials [23]. Grip strength (kg/BMI) was calculated as the mean of the maximum values of both hands [19,24] divided by the body mass index (BMI) [25]. In addition, grip strength was categorized into age-specific and sex-specific tertiles (upper, middle, and lower) to consider biological differences within sex and age groups (Supplementary Material 1).

### Type 2 diabetes mellitus and glycemic control

T2DM was defined as a fasting plasma glucose level ≥126 mg/dL among individuals who had fasted for over 8 hours or an HbA1c level ≥ 6.5% [26]. HbA1c is generally used to diagnose T2DM because it indicates an individual’s glycemic status in the last 2-3 months. As such, it was used to confirm typical blood glucose levels in patients with diabetes [5]. Thus, this study defined glycemic control in individuals diagnosed with T2DM as an HbA1c level < 6.5% [27, 28].

### Covariates

The following confounding factors were adjusted: age, sex, educational status (elementary school, middle school, high school, ≥ undergraduate), household income (low, middle-low, middle-high, high), smoking status (current, former, never), alcohol intake (at least once per month in the most recent year, less than once per month in the most recent year, never), family history of T2DM (yes, no), obesity (normal: 18.5-22.9; overweight: 23.0-24.9; obese: ≥ 25.0 kg/m^2^) [29], hypertension, hypercholesterolemia, and insulin use (yes, no). Household income was categorized into age-specific and sex-specific quartiles based on equalized household income, which was calculated by dividing the monthly household income by the square root of the number of household members. In addition, the criteria for diagnosing hypertension and hypercholesterolemia were as follows: systolic blood pressure ≥140 mmHg or diastolic blood pressure ≥ 90 mmHg and total cholesterol ≥ 240 mg/dL or use of anti-hypercholesterolemic drugs, respectively. In addition, the use of insulin for glycemic control was reported by participants in the questionnaire [30].

### Statistical analysis

According to grip strength levels, continuous variables are presented as mean and standard error, while categorical variables are presented as frequencies and proportions (weighted %) to describe subjects’ characteristics. One-way analysis of variance and the chi-square test were used to analyze differences between the groups. Multivariate logistic regression was used to examine associations between grip strength and glycemic control in patients with diabetes, and the results are reported as odds ratios (ORs) and 95% confidence intervals (CIs). Multivariate logistic regression analysis was adjusted for age and sex in model 1, for potential confounders in model 2, and for the use of insulin in model 3. All analyses were performed using the highest grip strength group as a reference group. Statistical significance was set at p-value < 0.05. Statistical analyses were performed using SAS version 9.4 (SAS Institute Inc., Cary, NC, USA).

### Ethics statement

This study was approved by the Institutional Review Board of Seoul National University (No. E2105/002-007).

## RESULTS

Table 1 shows the characteristics of the participants based on the tertiles of grip strength. The proportion of participants with a low education status, low household income, obesity, hypertension, and insulin use were highest in the lowest tertile of grip strength, while those who finished undergraduate studies and had a family history of T2DM were most frequent in the middle tertile of grip strength. The proportions of patients with a high-level of household income and alcohol intake of more than once per month were highest in the upper tertile of grip strength.

Table 2 shows the results of the multivariate logistic regression analysis of associations between grip strength and glycemic control in patients with diabetes. The lowest and middle tertiles of grip strength had significant ORs for glycemic control (0.65; 95% CI, 0.45 to 0.93 and 0.72; 95% CI, 0.52 to 0.99, respectively), compared to the highest tertile of grip strength after adjustment for confounders. Additionally, after adjusting for insulin use, the OR for the lowest tertile of grip strength was 0.67 (95% CI, 0.47 to 0.97). Nevertheless, the linear trend across the tertiles of grip strength was statistically significant (p for trend=0.031), although the middle tertile of grip strength had a statistically insignificant association (OR, 0.73; 95% CI, 0.53 to 1.01). In the analyses that considered continuous variables (increment per 0.05 kg/BMI), a significant association was observed after adjusting for the confounders (OR, 1.03; 95% CI, 1.01 to 1.06).

**Figure 2.**
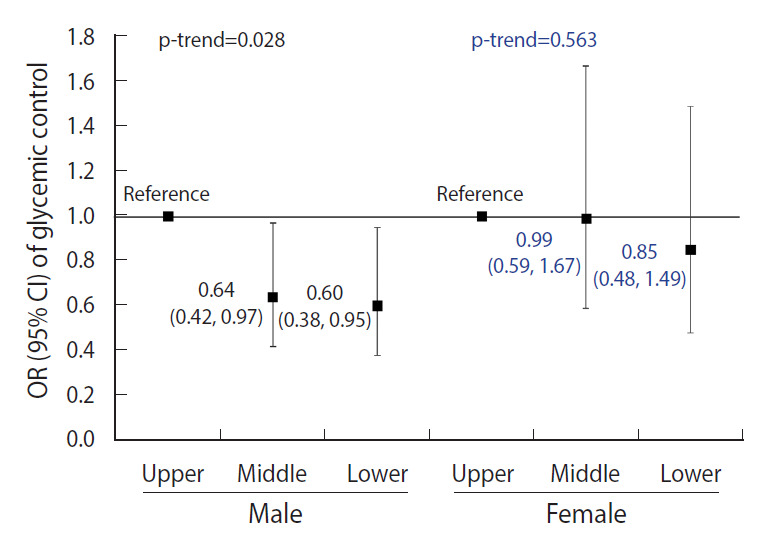
Subgroup analyses of the associations between grip strength and glycemic control in diabetes. Values are presented as OR (95% CI), adjusted for age, sex, education, house income, current smoking, alcohol intake, family history, obesity, hypertension, hypercholestrolemia, and insulin use. OR, odds ratio; CI, confidence interval.

Figure 2 shows the results of subgroup analyses based on sex. In male, the lower and middle tertiles of grip strength had statistically significant ORs for glycemic control (0.60; 95% CI, 0.38 to 0.95 and 0.64; 0.42 to 0.97 kg/m^2^, respectively) after adjusting for the confounders. However, no significant association between grip strength and glycemic control was observed in female.

## DISCUSSION

This was the first study to analyze the association between grip strength and glycemic control in Korean adults with T2DM, including a large population aged 19-80 years. Multivariate logistic regression analyses showed that the participants in the lowest tertile of grip strength had a significantly lower probability of glycemic control after adjusting for all confounding variables, including insulin use.

These findings are inconsistent with those of earlier research [30,31]. A previous study analyzed the data of 1,058 participants aged over 40 years and reported that grip strength was associated with glycemic control after adjusting for covariates, such as biological, behavioral, dietary, educational, and socioeconomic variables (p=0.073 for linear trend). However, after adjusting for additional insulin use, no significant association was observed [30]. Similarly, a study with 768 T2DM patients found that a higher HbA1c level (≥ 8.0%) was not associated with weak grip strength after adjustment for confounders [31]. These differences may be due to the following reasons: the previous studies were performed using data from American or Japanese patients aged over 40 years; the dependent variable was an HbA1c above 7% as the criterion of poor glycemic control and a dichotomous criterion was used for weak grip strength (< 26 kg for male and < 18 kg for female); and relative grip strength was used in this study, but the previous studies used absolute grip strength for their analyses. However, other observational studies on patients with diabetes over 40 years have shown that poor glycemic control was associated with lower muscle quality and mass [31-33].

Furthermore, a subgroup analysis by sex was performed in this study. The results showed that the middle and lower tertiles of grip strength were associated with poor glycemic control only in male. These sex differences in our results may be explained by differences in body composition. Generally, healthy females have more risk factors facilitating insulin resistance, including less muscle mass and a higher level of lipids in muscle cells than males [34,35]. In addition, females have a lower percentage of muscle mass than males in the upper extremities [36,37]. Thus, grip strength may be a more suitable indicator for males.

Insulin resistance is the primary metabolic defect in T2DM patients, and most patients with diabetes have severe insulin resistance in skeletal muscles [11,38]. In addition, insulin resistance is negatively correlated with muscle strength, as has been well established in previous studies [39,40]. In general terms, by inhibiting the binding of insulin with its receptor, insulin resistance impairs glycogen synthesis by suppressing insulin’s stimulation of the glucose transport system in the muscles and inhibits the protein synthesis pathway, which is responsible for muscle hypertrophy [41,42]. Therefore, severe insulin resistance causes impaired glucose metabolism and muscle atrophy in patients with T2DM [41,42], leading to a decline in muscle strength [43]. Consequently, low muscle strength and insulin resistance likely have deleterious effects on each other.

Plasma glucose enters the muscle cell by the GLUT4 transporter, either through the IRS/PI3K/Akt pathway or through muscle contraction (i.e., through insulin-dependent and insulin-independent pathways) [44,45]. As mentioned above, in most patients with diabetes, the insulin-dependent pathway is affected by insulin resistance, but the insulin-independent pathway is not damaged and appears to maintain a normal function [46]. An experimental study consistently showed that GLUT4 levels increased by 40% in legs trained with resistance exercise compared to untrained legs [47]. Thus, increased GLUT4 levels can promote glucose disposal in trained skeletal muscles [44]. Although there is a lack of evidence, differentiation in the severity of insulin resistance and GLUT4 content in skeletal muscles likely contributes to glucose metabolism in T2DM.

Our study had the following limitations. First, because the KNHANES only includes data from the Korean population, these results may not be generalizable to other countries. Second, since this was a cross-sectional study, we could not determine the causal relationship between grip strength and glycemic control in patients with T2DM. Lastly, the duration of diabetes affecting glycemic control was not adjusted in the current study. A major strength of this study is that it was the first examination of the association between grip strength and glycemic control in approximately 2,500 patients with diabetes after excluding individuals diagnosed with other diseases (i.e., CVD and cancer) using data representative of Korean adults.

In conclusion, grip strength is valuable indicator in the clinical field due to its simplicity and inexpensiveness compared to other methods of measuring muscle strength. Thus, our findings suggest that lower grip strength could be a valuable indicator for identifying patients with diabetes who are at the highest risk of poor glycemic control, especially in male. However, further studies are needed to investigate the causal relationship between grip strength and glycemic control in diabetes in future cohort studies involving Korean adults.

## Figures and Tables

**Table 1.** Characteristics of participants in the KNHANES 2014-2019 according to grip strength tertiles

Characteristics	Grip strength			p-value
	Upper (n=834)	Middle (n=836)	Lower (n=828)	
Weighted n	854,815	850,579	824,702	
Age	56.71±0.52	56.87±0.55	57.64±0.58	0.463
Sex				0.965
Male	436 (58.1)	438 (57.9)	433 (57.4)	
Female	398 (41.9)	398 (42.1)	395 (42.6)	
Education				0.003
Elementary school	227 (21.5)	262 (24.1)	291 (29.1)	
Middle school	131 (15.8)	131 (15.2)	138 (14.9)	
High school	301 (40.1)	262 (34.7)	220 (29.3)	
Undergraduate	175 (22.5)	181 (26.0)	179 (26.6)	
Household income				<0.001
Low	183 (17.3)	219 (23.3)	260 (27.3)	
Middle low	233 (26.8)	242 (29.3)	227 (27.7)	
Middle high	210 (28.7)	186 (23.0)	182 (23.8)	
High	208 (27.2)	189 (24.5)	159 (21.2)	
Obesity				<0.001
Normal	326 (38.3)	160 (18.8)	85 (9.3)	
Overweight	235 (27.5)	222 (25.7)	134 (14.5)	
Obese	273 (34.2)	454 (55.5)	609 (76.2)	
Smoking				0.392
Never	429 (46.1)	442 (49.5)	434 (49.0)	
Former	236 (29.5)	220 (26.8)	208 (24.7)	
Current	169 (24.4)	174 (23.7)	186 (26.3)	
Alcohol				0.012
Never	111 (11.4)	131 (12.9)	161 (16.8)	
<Once/mo	272 (30.7)	263 (29.7)	288 (32.6)	
≥Once/mo	451 (58.0)	442 (57.4)	379 (50.6)	
Family history				0.465
Yes	234 (31.7)	258 (35.0)	233 (32.7)	
No	600 (68.3)	578 (65.0)	595 (67.3)	
Hypertension				<0.001
Yes	409 (45.3)	500 (55.9)	545 (62.2)	
No	425 (54.7)	336 (44.1)	283 (37.8)	
Hypercholesterolemia				0.246
Yes	342 (38.2)	324 (36.9)	335 (41.3)	
No	492 (61.8)	512 (63.1)	493 (58.7)	
Insulin use				0.007
Yes	33 (3.1)	38 (4.1)	59 (6.5)	
No	802 (96.9)	798 (95.9)	769 (93.5)	

Values are presented as mean±standard error or number (weighted %). KNHANES, Korea National Health and Nutrition Examination Survey.

**Table 2.** Associations between grip strength and glycemic control^1^

Grip strength	Total	Cases	Model 1	Model 2	Model 3
Upper	834	147	1.00 (reference)	1.00 (reference)	1.00 (reference)
Middle	836	118	0.75 (0.55, 1.02)	0.72 (0.52, 0.99)	0.73 (0.53, 1.01)
Lower	828	106	0.69 (0.50, 0.95)	0.65 (0.45, 0.93)	0.67 (0.47, 0.97)
p for trend			0.021	0.018	0.031
Increment per 0.05 kg/BMI			1.03 (1.01, 1.06)	1.04 (1.01, 1.07)	1.03 (1.01, 1.06)

Values are presented as odds ratio (95% confidence interval). BMI, body mass index.

1 Model 1 was adjusted for age and sex; Model 2 was adjusted for model 1 plus education, household income, current smoking, alcohol, family history, obesity, hypertension, and hypercholesterolemia; Model 3 was adjusted for model 2 plus insulin use.
