# Supplementary material for: Associations between grip strength and glycemic control in type 2 diabetes mellitus: an analysis of data from the 2014-2019 Korea National Health and Nutrition Examination Survey
Source: Epidemiol Health. 2021 Oct 8;43:e2021080. doi: 10.4178/epih.e2021080 (PMC8859497; doi:10.4178/epih.e2021080)
Supplement: Supplementary file 1 [file epih-43-e2021080-suppl.docx]

SUPPLEMENTARY MATERIAL

Supplementary Material 1. The cut value of the grip strength according to the sex and age group

| Sex | Age group | Lower | Middle | Upper |
| --- | --- | --- | --- | --- |
| Men | 19-29 | <1.21 | 1.21-1.39 | >1.39 |
|  | 30-39 | <1.41 | 1.41-1.64 | >1.64 |
|  | 40-49 | <1.46 | 1.46-1.71 | >1.71 |
|  | 50-59 | <1.44 | 1.44-1.66 | >1.66 |
|  | 60-69 | <1.36 | 1.36-1.61 | >1.61 |
|  | over 70 | <1.17 | 1.17-1.41 | >1.41 |
|  |  |  |  |  |
| Women | 19-29 | <0.75 | 0.75-0.91 | >0.91 |
|  | 30-39 | <0.84 | 0.84-1.01 | >1.01 |
|  | 40-49 | <0.84 | 0.84-1.03 | >1.03 |
|  | 50-59 | <0.83 | 0.83-1.02 | >1.02 |
|  | 60-69 | <0.77 | 0.77-0.95 | >0.95 |
|  | over 70 | <0.67 | 0.67-0.83 | >0.83 |

Values are presented as kg/BMI.
